# *Pseudomonas aeruginosa* zinc uptake in chelating environment is primarily mediated by the metallophore pseudopaline

**DOI:** 10.1038/s41598-017-16765-9

**Published:** 2017-12-07

**Authors:** Sébastien Lhospice, Nicolas Oswaldo Gomez, Laurent Ouerdane, Catherine Brutesco, Ghassan Ghssein, Christine Hajjar, Ahmed Liratni, Shuanglong Wang, Pierre Richaud, Sophie Bleves, Geneviève Ball, Elise Borezée-Durant, Ryszard Lobinski, David Pignol, Pascal Arnoux, Romé Voulhoux

**Affiliations:** 1 CNRS et Aix-Marseille Université, Laboratoire d’Ingénierie des Systèmes Macromoléculaires (UMR7255), Institut de Microbiologie de la Méditerranée, Marseille, France; 2 Université de Pau et des Pays de l’Adour/CNRS, Laboratoire de Chimie Analytique Bio-inorganique et Environnement, IPREM-UMR5254, Hélioparc, 2, Avenue Angot, 64053 Pau, France; 3 CEA, CNRS and Aix-Marseille Université, Institut de Biosciences et Biotechnologies d’Aix-Marseille, UMR 7265 LBC, CEA Cadarache, Saint-Paul-lez-Durance, F-13108 France; 4 CEA, CNRS and Aix-Marseille Université, Institut de Biosciences et Biotechnologies d’Aix-Marseille, UMR 7265 LB3M, CEA Cadarache, Saint-Paul-lez Durance, F-13108 France; 5 Micalis Institute, INRA, AgroParisTech, Université Paris-Saclay, 78350 Jouy-en-Josas, France

## Abstract

Metal uptake is vital for all living organisms. In metal scarce conditions a common bacterial strategy consists in the biosynthesis of metallophores, their export in the extracellular medium and the recovery of a metal-metallophore complex through dedicated membrane transporters. Staphylopine is a recently described metallophore distantly related to plant nicotianamine that contributes to the broad-spectrum metal uptake capabilities of *Staphylococcus aureus*. Here we characterize a four-gene operon (*PA4837*–*PA4834*) in *Pseudomonas aeruginosa* involved in the biosynthesis and trafficking of a staphylopine-like metallophore named pseudopaline. Pseudopaline differs from staphylopine with regard to the stereochemistry of its histidine moiety associated with an alpha ketoglutarate moiety instead of pyruvate. *In vivo*, the pseudopaline operon is regulated by zinc through the Zur repressor. The pseudopaline system is involved in nickel uptake in poor media, and, most importantly, in zinc uptake in metal scarce conditions mimicking a chelating environment, thus reconciling the regulation of the *cnt* operon by zinc with its function as the main zinc importer under these metal scarce conditions.

## Introduction

Divalent metals (Mn, Fe, Co, Ni, Cu and Zn) are essential micronutrients for all life forms, and acquisition of these metals is therefore vital, particularly for bacterial pathogens in the context of host-pathogen interactions. Indeed, there is a competition between the host, which tends to sequester metals in a so called nutritional immunity framework, and the pathogenic bacterium, which increases its metal uptake efforts in order to keep up with its metal requirements^1,2^. Most pathogenic bacteria produce metallophores for metal uptake, with siderophores being the most well-characterized metallophore family^3^. Siderophores are synthesized within the cell through non ribosomal peptide synthases (NRPS) or through a NRPS independent system (NIS) and then are exported in the extracellular medium where they scavenge iron. Extracellular iron siderophore complexes can be recognized and actively transported into the periplasm by TonB dependent transporters (TBDT) in Gram-negative bacteria, and usually ABC transporters in both Gram-negative and Gram-positive bacteria are used to transport the metals across the cytoplasmic membrane. There are many variations on this common theme, with some bacteria unable to produce specific siderophore but capable of scavenging them from the environment for iron import^4^. The siderophore pathway could also prevent toxic accumulation of metals, which has specifically been investigated in the case of *Pseudomonas aeruginosa* ^5,6^. *P. aeruginosa* synthesizes two types of siderophores with high iron affinity (pyochelin and pyoverdine) the latter being a demonstrated virulence factor^7^.

Metallophores specific for the uptake of metals other than iron have also been described, such as the chalcophore methanobactin involved in copper uptake in methane-oxidizing bacteria^8,9^. Manganesophores have not been described as such, although TseM, a protein effector secreted through a Type VI secretion system, was shown to play an important role in TBDT-dependent manganese uptake in *Burkholderia thailandensis* ^10^. There is also indirect evidence for the existence of a nickelophore in *Escherichia coli*, although it has still to be identified^11^. Free histidine could also be used as a nickelophore *in vivo* for nickel uptake in various bacteria^12,13^. Yersiniabactin, initially described as a siderophore, also exhibits zincophore properties in *Yersinia pestis* ^14,15^. Coelibactin, described in *Streptomyces coelicolor*, may also represent a zincophore as it is synthesized by a NRPS under the control of Zur, a zinc responsive repressor^16^.

Staphylopine is a nicotianamine-like molecule that was recently described as a metallophore with remarkable broad-spectrum specificity^17^. In *Staphylococcus aureus*, staphylopine is synthesized through the action of three soluble enzymes (SaCntKLM). SaCntK transforms L-histidine in D-histidine, SaCntL transfers an aminobutyrate moiety from S-adenosylmethionine (SAM) onto D-histidine, and SaCntM reductively condensates the product of SaCntL (called xNA) with pyruvate. Depending on the growth conditions, the staphylopine biosynthesis and trafficking pathway is responsible for zinc, copper, nickel, cobalt and iron uptake, and this system contributes to the virulence and fitness of *S. aureus* ^17–19^. The *S. aureus cnt* operon is partly conserved in *P. aeruginosa*, where homologues of the *cntL* and *cntM* genes are found, albeit with 20–30% sequence identity at the protein level. Upstream of *cntL*, a gene codes a predicted outer membrane protein belonging to the TBDT family (here named *cntO;* see Supplementary Table S1 for correspondence with locus tag in PAO1, PA7 and PA14 strains of *P. aeruginosa*), and downstream of *cntM*, a gene codes for a predicted inner membrane protein belonging to the EamA or DMT family (drug/metabolite transporter; here named *cntI*, see Supplementary Table. 1). Transcriptomic approaches revealed that this gene cluster was highly expressed in a burn wound model^20^. This last gene was also identified as part of a novel siderophore pathway that appeared vital for the growth of *P. aeruginosa* in airway mucus secretion (AMS)^21^. Finally, through a transcriptomic study of a Znu deficient strain, these four genes were found in the top five regulated units, although most of them were annotated as hypothetical^22^.

Here, we show that the four above-mentioned genes *cntOLMI* are part of an operon that is negatively regulated by zinc level through the Zur repressor. Using biochemical and metabolomic approaches, we demonstrate that the two biosynthetic enzymes (PaCntL and PaCntM) synthesize a novel metallophore, which we named pseudopaline. Pseudopaline differs from staphylopine by the presence of a L-histidine moiety instead of D-histidine, and an α-ketoglutarate moiety instead of a pyruvate. A *cntL* mutant strain was shown to be unable to synthesize pseudopaline and was impaired in its ability to import nickel in a minimal media, supplemented or not with nickel. Under more stringent conditions where a chelator such as EDTA was added to a minimal succinate (MS) medium, a condition that presumably mimics the chelating environment prevailing within a host or in AMS, we showed evidence that the *cntL* mutant strain was unable to import zinc, therefore reconciling the regulation of this operon by zinc with its function as a zinc importer operating in metal scarce conditions.

## Results and Discussion

### The *cnt* operon of *P. aeruginosa* is regulated by zinc level through the zinc-responsive regulator Zur

**Figure 1:**
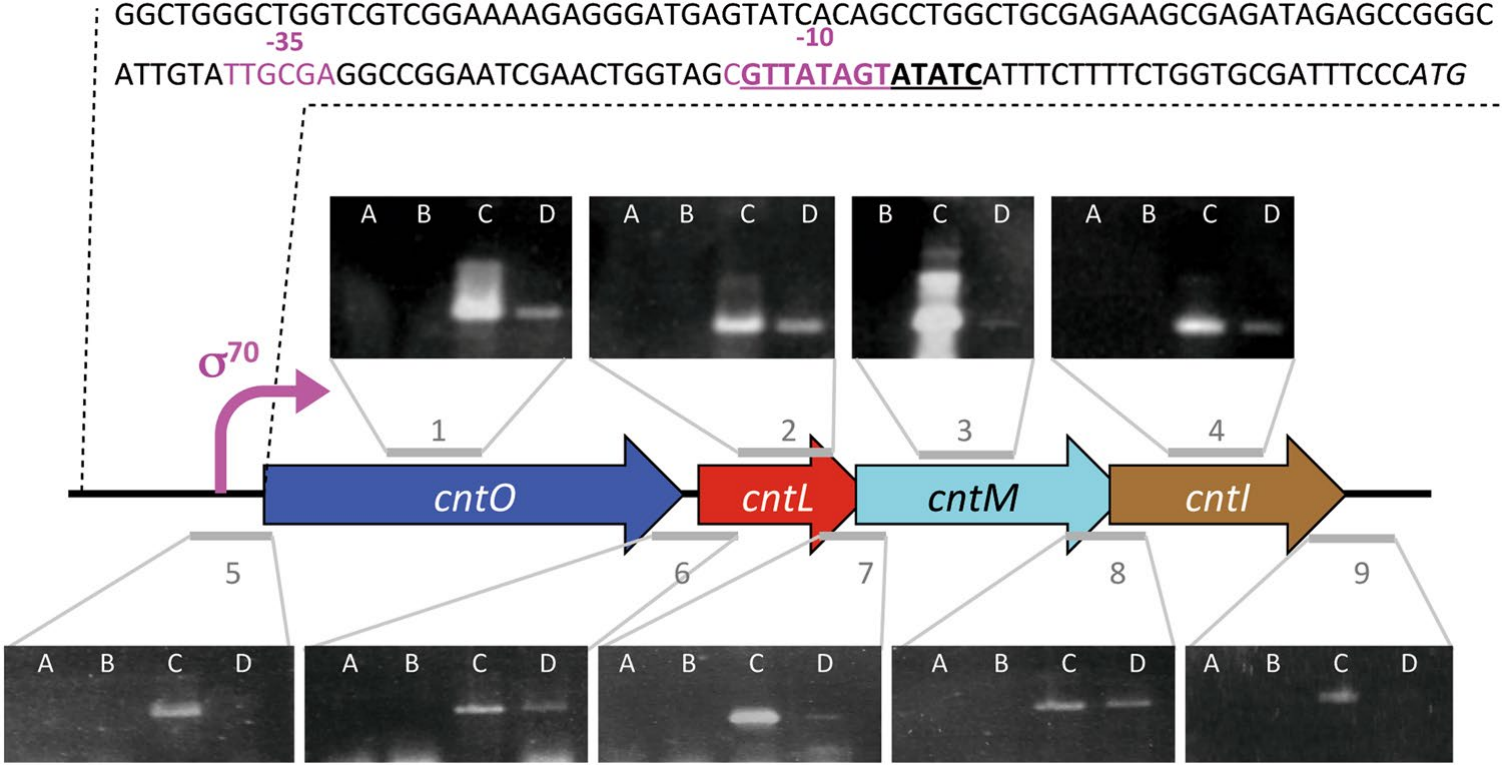
Determination of the *cnt* operon of *Pseudomonas aeruginosa*. Schematic representation of the genetic environment of the four *cnt* genes recovered on the *P. aeruginosa* PA14 genome. The upstream DNA sequence carrying the −35 and −10 boxes of the σ 70 promoter are highlighted in pink and the predicted palindromic *Zur bo*x is underlined. The first initiating *ATG* codon of *cntO* gene is indicated in italic. RT-PCR were performed on total RNA isolated from *P. aeruginosa* PA14 strain grown in MS medium. PCR were performed without template (**A**) or with RNA (**B**), genomic DNA (**C**) or cDNA (**D**) as template. PCR corresponding to the four intragenic regions (1 to 4) and the five intergenic regions (5 to 9) tested are indicated in gray.

*In silico* analysis of the *cnt* gene cluster of *P. aeruginosa* PA14 strain indicated two overlapping open reading frames between *cntL* and *cntM* and between *cntM* and *cntI*, classically observed in operonic structures (Fig. 1). Further screening of the upstream *cnt* sequence for promoter regions using Bprom software^23^, revealed a σ70 promoter in the 200 base-pairs upstream from the annotated *cntO* ATG codon (Fig. 1). As already reported by Pederick and collaborators, a putative Zur binding box “GTTATagtATAtC” overlaps the −10 box of the predicted σ70 promoter^22,24^. This *in silico* analysis supports an operonic organization of the four *cnt* genes and strongly suggests a transcriptional activation of this operon under zinc depletion through the Zur repressor^25,26^. In order to test this hypothesis we performed RT-PCR experiments using as templates RNA and cDNA generated from a WT PA14 strain grown in minimal succinate (MS) medium known to contains low levels of metals, including zinc^5^. The successful amplification of the four *cnt* gene transcripts (Fig. 1) indicated their induction when cells were grown in a MS medium. The specific amplification of the three *cnt* intergenic regions confirmed that the four *cnt* genes are co-transcribed in one single transcript and therefore constitute an operon.

**Figure 2:**
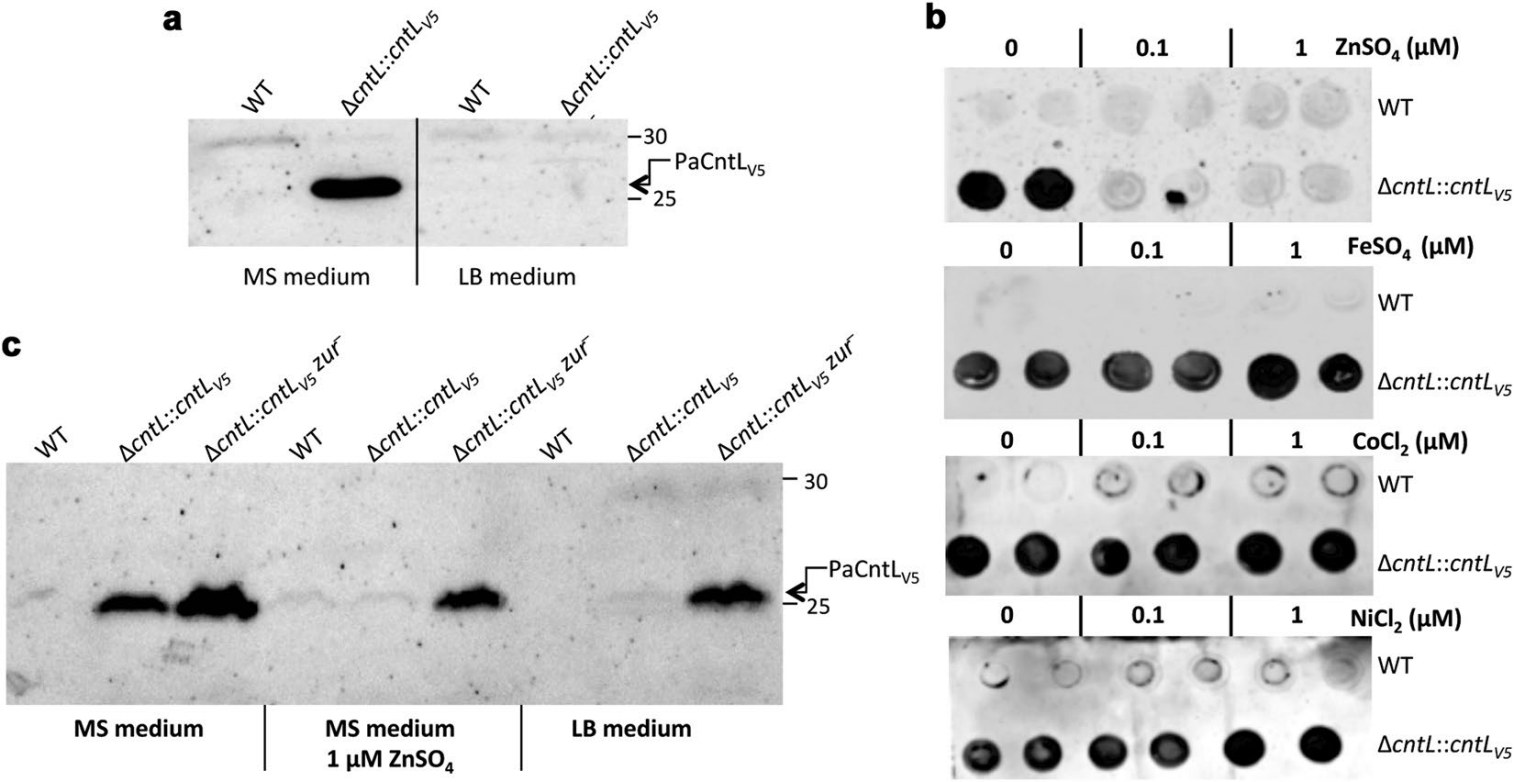
PaCntL production under various growth conditions. **(a)** Immunoblotting using antibody directed against the V5 epitope for revealing PaCntL_V5_ production under poor (MS) and rich (LB) media. (**b**) Dot-blot revealing the PaCntL_V5_ production in MS medium supplemented by divalent metals. (**c**) Immunoblot detection of PaCntL_V5_ production in PA14 WT and Zur deficient strains (*zur* ^*−*^) in various growth conditions.

To validate at the protein level the transcriptional regulation of the *cnt* genes, we followed the PaCntL production under various growth conditions by immunoblotting. In this respect, we constructed a *cntL* mutant strain producing a chromosomally encoded V5-tagged PaCntL (∆*cntL::cntL*_*V5*_). In this strain the recombinant *cntL*_V5_ gene was placed under the predicted *cnt* promoter region and inserted at the *att* site of the *P. aeruginosa* genome. In agreement with our transcriptional data, immunoblotting experiments indicated that, the recombinant PaCntL_V5_ is only produced in MS medium and not in a rich medium such as the LB medium (Fig. 2a). We then tested whether the *cntL* transcription was subject to metal repression by checking PaCntL_V5_ production in MS medium supplemented with various concentrations of divalent transition metal ions. Dot-blot experiments showed a specific loss of PaCntL_V5_ production in MS medium supplemented with as low as 0.1 μM of ZnSO_4_. An addition of iron, nickel or cobalt at concentrations equivalent or above the one found in LB rich medium^5^ has no negative effect on PaCntL_V5_ production (Fig. 2b). In the same line, copper was not a negative repressor of PaCntL_V5_ expression (data not shown). The hypothesis of a Zur repressor regulating the *cnt* operon was then tested through the construction of a PA14∆*cntL::cntL*_*V5*_ *zur* ^*−*^ strain. PaCntL_V5_ was still produced in the *zur* mutant strain grown in LB or MS media supplemented with 1 μM of ZnSO_4_, conditions in which Zur normally exerts its repressor activity (Fig. 2c). Overall, these data demonstrate that the regulation of the *cnt* operon of *P. aeruginosa* by zinc is exerted by the Zur repressor complex, most probably through its binding onto the predicted Zur binding motif identified in the σ70 promoter.

### *In vivo* detection and characterization of a PaCntL-dependent metallophore in the extracellular medium of *P. aeruginosa*

**Figure 3:**
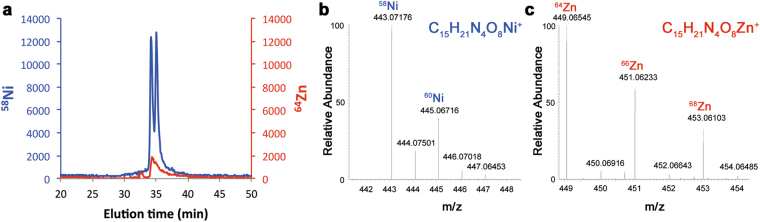
*In vivo* PaCntL-dependent detection of a nickel or zinc-bound metallophore in the extracellular fraction of *P. aeuginosa*. **(a)** HILIC/ICP-MS chromatogram of metal-bound metabolites found in the extracellular fraction of P. aeruginosa grown in MS medium. (**b**) HILIC-ESI/MS mass spectrum of a Ni-metallophore complex in the extracellular fraction of the WT strain but absent in the Δ*cntL* mutant (both grown in MS medium). (**c**) HILIC-ESI/MS mass spectrum of a Zn-metallophore complex in the extracellular fraction of the WT strain but absent in the Δ*cntL* mutant. The empirical molecular formula of the CntL-dependent Ni- or Zn-metallophore complexes were deduced from the exact mass.

We constructed a PA14 mutant strain lacking PaCntL (Δ*cntL*) and compared the composition of the intra- and extra-cellular contents of wild type and Δ*cntL* strains grown under the previously defined *cnt* inducible conditions. Extracellular samples were analysed by hydrophilic interaction liquid chromatography (HILIC) with detection by inductively coupled plasma mass spectrometry (ICP-MS) and electrospray ionization mass spectrometry (ESI-MS). HILIC/ICP-MS data revealed the presence of a molecule complexed with nickel and zinc in the supernatant of the WT strain, which was absent in the *cntL* mutant strain (Fig. 3). ESI-MS investigation of the metabolites eluting at this same elution volume showed unambiguously the presence of typical nickel and zinc isotopic patterns indicating the presence of a free metallophore with a molecular mass of 386 Da (Fig. 3). Using the accurate mass and a molecular formula finder software we proposed the C_15_H_20_N_4_O_8_ empiric formula for the ligand in complex with nickel or zinc (Fig. 3). This ligand corresponds to a new metallophore produced by *P. aeruginosa* in a *cntL*-dependent manner. Comparison of its elemental composition with that of staphylopine (328 Da) revealed the presence of two additional carbons and two oxygen atoms, suggesting the use of an α-ketoglutarate (αKG) moiety instead of pyruvate as found in staphylopine. The fragmentation of this metallophore in gas-phase confirmed this hypothesis (Supplementary Fig. 1). We propose to name this new metallophore pseudopaline, to recall its origin from *P. aeruginosa* and its belonging to the nopaline family of opine^27^.

### *In vitro* reconstitution of the pseudopaline biosynthetic pathway catalysed by PaCntL and PaCntM

**Figure 4:**
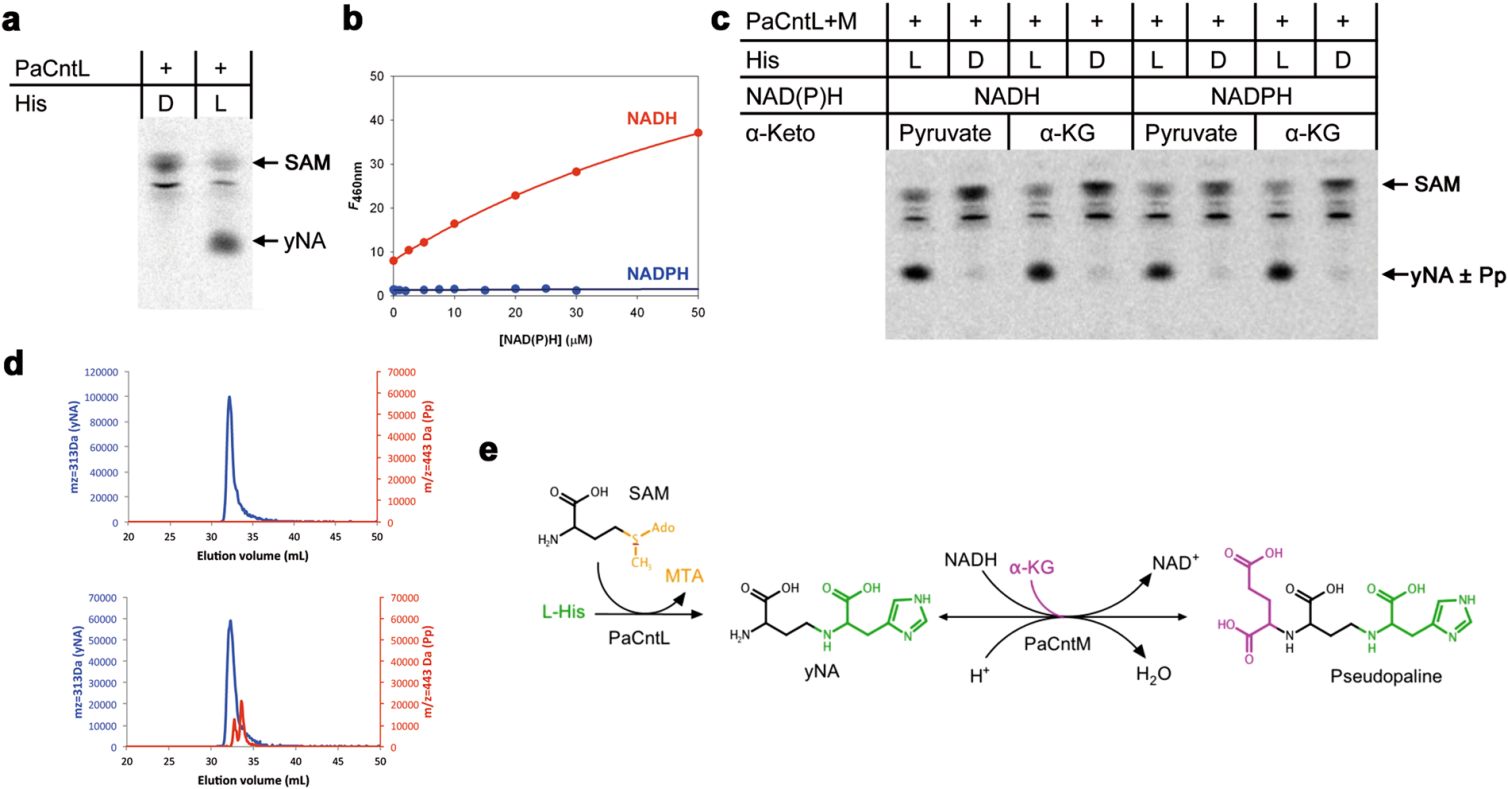
*In vitro* reconstitution of the pseudopaline biosynthesis pathway. **(a)** TLC experiment using PaCntL and [^14^C]-SAM showing that PaCntL discriminates between D- and L-histidine substrate with the production of the reaction intermediate (noted yNA) only visible when using L-histidine. (**b**) Titration of NADPH (blue) and NADH (red) binding to PaCntM (5 µM) followed by fluorescence resonance energy transfer. Fitting of the data obtained for NADH led to a K_d_ of 30 µM. (**c**) TLC separation of reaction products after incubation of [^14^C]-SAM with purified enzymes (PaCntL and PaCntM), different source of α-ketoacid (pyruvate or α-KG), cofactor (NADH or NADPH) and histidine (L-His or D-His). (**d**) HILIC/ESI-MS chromatograms of putative reaction products using PaCntL incubated with L-histidine, revealing the production of the yNA intermediate (top), and a mix of PaCntL and PaCntM incubated with all their putative substrate (SAM, L-histidine, NADH and α-Ketaoglutarate), revealing the specific detection of pseudopaline in this case (red trace, bottom). (**e**) Summary of the PaCntL/M-dependent biosynthesis pathway for the assembly of pseudopaline from L-his, SAM, NADH and α-KG.

We have recently shown that the PaCntL/M orthologs in *S. aureus* (SaCntL/M) are sequentially involved in the biosynthesis of the staphylopine metallophore, using a D-histidine that is produced by the histidine racemase enzyme SaCntK^17^. One of the main difference between the *cnt* operons of *P. aeruginosa* and *S. aureus* is the absence of a *cntK* gene upstream of the *cntL-M* genes in *P. aeruginosa*. This observation led to the possibility of using directly L-histidine instead of D-histidine. In order to investigate the properties of CntL and CntM of *P. aeruginosa*, the corresponding genes were cloned, heterologously expressed in *E. coli* and their products purified for further biochemical analysis. Gel filtration experiments showed that PaCntL could form a complex with PaCntM (Supplementary Fig. 2), although this interaction was not observed between SaCntL and SaCntM. With regard to PaCntL, we used thin layer chromatography (TLC) separation to follow the carboxyl moiety of a carboxyl-[^14^C]-labelled S-adenosine methionine (SAM) substrate, co-incubated with either L- or D-histidine (Fig. 4a). Only the incubation with L-histidine led to a novel band corresponding to a reaction intermediate that we propose to name yNA. We demonstrated subsequently that PaCntM preferentially bound to NADH and not to NADPH (Fig. 4b), contrary to SaCntM that showed a preference for NADPH. We then used TLC to visualize the PaCntLM reaction products under various *in vitro* conditions using all the putative substrates (Fig. 4c). Unexpectedly, the co-incubation of both enzymes with their most probable substrates (L-histidine, NADH and αKG) did not lead to the formation of an additional radiolabelled product as for the case of staphylopine biosynthesis^17^ (Fig. 4c). One possibility was therefore that the product of PaCntM was migrating at the same position as the yNA in the conditions used during the TLC separation. We therefore decided to study the same co-incubations by HILIC/ESI-MS, following the mass expected for the yNA intermediate and the pseudopaline found in the extracellular fraction of *P. aeruginosa* grown in MS medium. These experiments confirmed that the incubation of PaCntL with SAM and L-histidine led to the formation of the yNA reaction intermediate (Fig. 4d, top), and most importantly, revealed the production of pseudopaline when co-incubating PaCntL, PaCntM and their proposed substrates (SAM, L-histidine, NADH and αKG; Fig. 4d, bottom). Co-incubations using alternative substrates of PaCntM (pyruvate or NADPH) only led to the production of yNA. Interestingly, pseudopaline and yNA eluted at the same volume in these HILIC-ESI/MS experiments, showing that their physical properties are very similar, as suggested by our previous TLC experiments.

Pseudopaline is therefore biosynthesized in two steps: first, a nucleophylic attack of one α-aminobutyric acid moiety from SAM onto L-histidine catalysed by PaCntL to produce the reaction intermediate yNA, and second, a NADH reductive condensation of the yNA intermediate with a molecule of αKG catalysed by PaCntM to produce pseudopaline (Fig. 4e). Pseudopaline differs from staphylopine by the stereochemistry of the histidine moiety (L- and D- respectively) and by the presence of an αKG moiety instead of pyruvate in staphylopine. The biosynthesis of a specific metallophore by different bacteria recalls the chemical evolution of a large diversity of siderophore in a chemical rivalry to get access to one’s own pool of metal^28^. Indeed, once in the extracellular medium, secreted metallophores are a common good, and a privileged access presumably gives a selective advantage.

### Pseudopaline is involved in nickel and zinc uptake, depending on the chelating properties of the media

**Figure 5:**
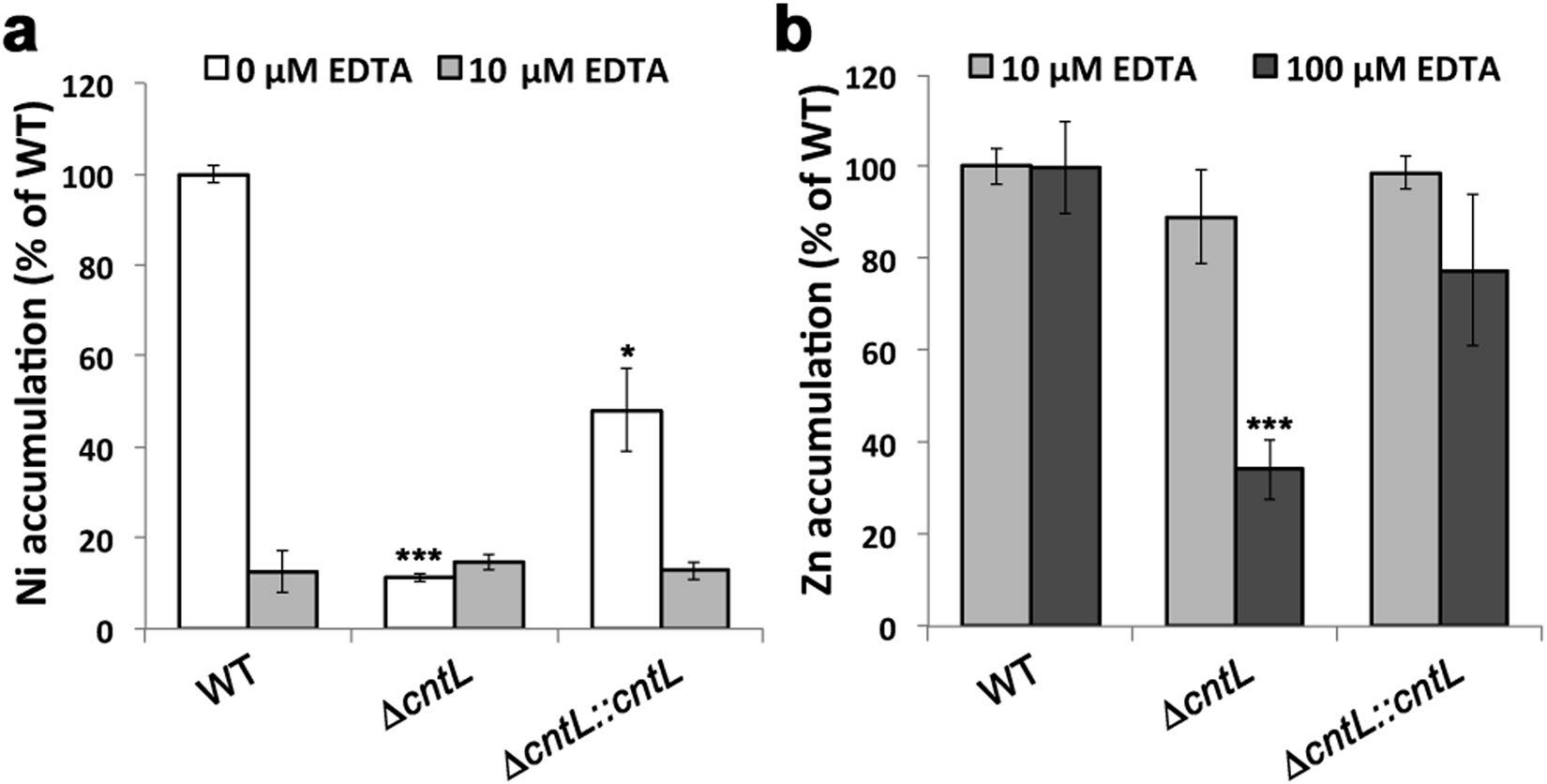
Pseudopaline is involved in nickel uptake in minimal media and in zinc uptake in chelating media. Intracellular nickel (**a**) or zinc (**b**) levels measured by ICP-MS in WT, *ΔcntL* and *ΔcntL::cntL* strains grown in MS medium supplemented or not with 10 or 100 µM EDTA. Error bars, mean ± s.d. *P < 0.05, **P < 0.01 and ***P < 0.001 as compared to the WT.

In order to address the involvement of pseudopaline in metal uptake *in vivo*, we compared the intracellular concentration of various metals in PA14 WT, *ΔcntL* and *ΔcntL::cntL* strains. Cells were grown in pseudopaline-synthesis conditions determined above (MS medium) and the intracellular metal concentration was measured by ICP-MS. Under these growth conditions we observed a significant 90% reduction of intracellular nickel concentration in the *ΔcntL* mutant strain (Fig. 5a), which was mostly recovered in the complemented strain. The levels of all the other metals were not changed in the *ΔcntL* mutant strain compared to the WT strain (data not shown). A similar 90% reduction in intracellular nickel concentration was also observed when the culture was supplemented with 1 μM NiCl_2_ (Supplementary Fig. 3), thus confirming that nickel uptake was predominantly performed by pseudopaline in these metal-poor media. Understanding whether pseudopaline-mediated nickel uptake is beneficial for the bacteria requires further investigations. We were however intrigued by the apparent contradiction between the clear *cnt* operon regulation by zinc, and the absence of any effect on zinc uptake. A possible explanation is that the effect of *cnt* could be masked by the effect of a free zinc ion importer such as the ZnuABC zinc transport system described in *P. aeruginosa* ^22,29^. In an attempt to discriminate between both transport systems, we sequestered free metal ions by supplementing the growth medium with increasing concentrations of EDTA, a chelating agent for divalent metals. Interestingly, although we did not observe any effect using 10 μM EDTA, the supplementation with 100 μM EDTA ultimately revealed a pseudopaline-dependent zinc uptake, with a 60% decrease of intracellular zinc content in the *ΔcntL* mutant strain in comparison with the WT strain (Fig. 5b). The complemented strain accumulated zinc to a level comparable to the WT. When using as low as 10 μM EDTA, the pseudopaline-dependent nickel import is not visible anymore (Fig. 5a), hence proving a direct link between pseudopaline and zinc uptake in metal scarce conditions containing competing zinc chelators. These conditions may prevail at the host-pathogen interface where metal binding proteins such as calprotectin are produced by the host^30,31^, or in AMS where metals are complexed in a nutritional immunity framework^1,21^.

### Model of pseudopaline synthesis and transport pathway in *P. aeruginosa*

**Figure 6:**
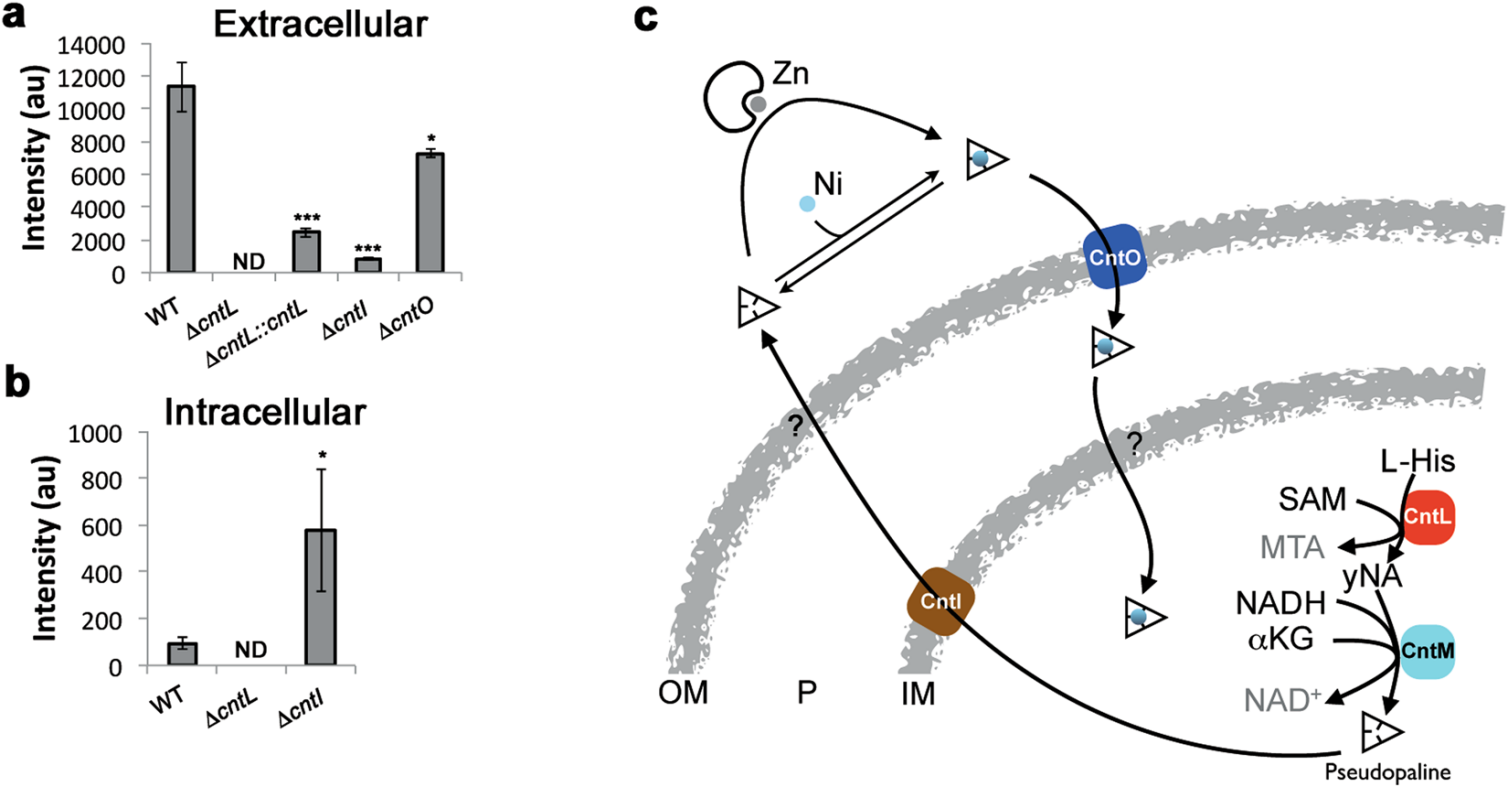
Model of pseudopaline synthesis, secretion and metal uptake in *P. aeruginosa*. **(a)** Detection of pseudopaline in the extracellular fraction of WT and mutant strains grown in MS medium. Error bars, mean ± s.d. *P < 0.05, **P < 0.01 and ***P < 0.001 as compared to the WT. (**b**) Detection of pseudopaline in the intracellular fraction of WT and mutant strains. ND: Not Detectable. (**c**) Model of pseudopaline production, secretion and recovery of nickel or zinc. Outer membrane (OM), inner membrane (IM), periplasm (P).

We next investigated the putative roles of the two membrane proteins that are found in the *cnt* operon of *P. aeruginosa* by determining the pseudopaline level in the extracellular and intracellular fractions of WT and mutant strains (Fig. 6a and b, respectively). With regard to PaCntO, we found a small decrease in the extracellular content of pseudopaline in the Δ*cntO* mutant strain in comparison with the WT strain. However, we also found that this Δ*cntO* mutant strain was partly impaired in nickel accumulation (Supplementary Fig. 4). Altogether, and because PaCntO belongs to the TBDT family of extracellular transporter, its most probable role is in the import of pseudopaline-metal complexes, although it is not excluded that other proteins of this family could participate in this process. Next, we noted a large decrease in the extracellular pseudopaline level in the Δ*cntI* mutant strain in comparison with the WT strain, with a concomitant increase in the intracellular space, consistent with a role of PaCntI in pseudopaline export. A model recapitulating the pseudopaline pathway is shown in Fig. 6. It is interesting to note that a Δ*cntI* mutant strain is unable to grow in AMS and impaired in iron accumulation in this media supplemented with iron^21^. Here we reveal an essential role of CntI in the export of pseudopaline, raising questions about its link with iron uptake. In MS medium, where the two major siderophores are active^5^, we did not found the essential function of the *cnt* operon in iron uptake. A few scenarios could be envisioned here, all coming from the observed pseudopaline accumulation which coincide with this *cntI* mutation: (*i*) accumulation of a metal chelator such as pseudopaline within the cytoplasm, might destabilize all the intracellular metal homeostasis by chelating metals already present within the cell. This assumption is supported by our finding that a double Δ*cntL*Δ*cntI* mutant suppresses the detrimental growth defect of the single Δ*cntI* mutant strain, *i.e*. the absence of pseudopaline restores the normal growth of a mutant devoid of the pseudopaline exporter (Supplementary Fig. 5), (*ii*) the intracellular pseudopaline accumulation might actually modify the zinc import in AMS (as shown here in a chelated MS medium) and, in turn, modify iron homeostasis through a dual or overlapping iron/zinc regulation, (*iii*) a zinc dependent enzyme might be involved in iron homeostasis, (*iv*) pseudopaline might be involved in ferrous iron transport in AMS and this effect might be masked in MS medium where iron is predominantly in its ferric state. A combination of all these scenarios is also possible and demands further investigations of this complex phenotype of the *cntI* mutant in various growth conditions.

It is interesting to note the differences and similarities between staphylopine and pseudopaline and between their respective biosynthetic pathways (Supplementary Fig. 6). On one hand, pseudopaline differs from staphylopine by the incorporation of a L-histidine instead of a D-histidine moiety in staphylopine, thus explaining the absence of amino acid racemase in *P. aeruginosa*. Another particularity of pseudopaline is the use of an αKG moiety instead of pyruvate as substrate for the second reaction mediated by PaCntM. Together this leads to two species-specific metallophores that might give a selective advantage in a competing environment. Either pyruvate or αKG are central molecules of global bacterial metabolism and it would therefore be interesting to investigate the effect of known metabolic regulators such as glucose on the modulation of the production of these metallophores. The fact that staphylopine and pseudopaline belong to Gram-positive and Gram-negative bacteria, respectively, has important consequences on their transport pathways across the bacterial membranes into the cytoplasm. Although the transporters of staphylopine are well identified, the outer membrane exporter pseudopaline and inner membrane importer of the pseudopaline-metal complex remains to be discovered (Fig. 6). Recycling of the metallophore could also take place in *P. aeruginosa*, as recently exemplified in the case for pyoverdine^32^. An interesting aspect of this work is the discovery of two different pathways for the export of these nicotianamine-like bacterial metallophores. Whereas *S. aureus* uses a protein belonging to the MFS family (SaCntE) for staphylopine export, *P. aeruginosa* uses a protein belonging to the DMT family of transporters, with PaCntI possessing two predicted EamA domains for pseudopaline export. In the view of the importance of metallophores such as pseudopaline and staphylopine in the growth or virulence of human pathogens^19,21^, these molecules could emerge as attractive targets for novel antimicrobial development.

### Note added in proof

During the revision of this manuscript, Mastropasqua and colleagues published an article demonstrating that the *cnt* operon (called *zrm* in their case, we favor the *cnt* naming convention because of the sequence conservation and functional similarities of the two biosynthetic genes between various bacterial operons) is induced under zinc depletion^33^. Moreover, and in agreement with our findings, they show the involvement of CntO in zinc uptake as well as its contribution to bacterial growth in zinc poor media. In addition, the authors observed a reduction in intracellular cobalt content, back to the WT level, between a *znuA* and a *znuA*/*cntO* mutant strain, suggesting that the pseudopaline pathway promotes the uptake of cobalt in bacteria unable to import zinc through ZnuABC. Using a double *cntOL* mutant, they further show that the outer membrane protein CntO is the receptor of a putative zincophore, that we called pseudopaline and fully characterized in the present study.

## Materials and Methods

### Bacterial strains, plasmids and growth conditions

Bacterial strains, vectors and plasmids used in this study are listed in Supplementary Table 2. *E. coli* strains were grown aerobically with shaking at 37 °C in Luria-Broth (LB) with antibiotics as required (50 μg ml^−1^ ampicillin (Ap), 25 μg ml^−1^ kanamycin (Kan), 25 μg ml^−1^ tetracycline (Tc), 15 μg ml^−1^ gentamicin (Gm), 30 μg ml^−1^ streptomycin (Sm)). The *E. coli* strains CC118λ*pir* and SM10 were respectively used to propagate pKNG101 derivatives mutator plasmids and Mini-CTX1 plasmids. Recombinant plasmids were introduced in *P. aeruginosa* by triparental mating using pRK2013 and transconjugants selected on *Pseudomonas* isolation agar (PIA, Difco Laboratories) supplemented with antibiotics as required (500 μg ml^−1^ carbenicillin (Cb), 150 μg ml^−1^ Gm, 2000 μg ml^−1^ Sm, 200 μg ml^−1^ Tc). All the *P. aeruginosa* strains used in this study were derivatives of the parental PA14 strain. *P. aeruginosa* strains were grown aerobically with horizontal shaking at 37 °C with antibiotics as required (150 μg ml^−1^ Cb, 50 μg ml^−1^ Gm, 500 μg ml^−1^ Sm, 50 μg ml^−1^ Tc). Growths were performed in TSB rich medium (Difco), minimal succinate (MS) medium^34^ or chemically defined media (CDM)^18^. When specified, Nickel (NiCl_2_ 6H_2_O), or Ethylenediaminetetraacetic acid (EDTA) were added to the media. Growth was monitored by OD600 measurement.

### Plasmid construction

All plasmids constructed in this study were obtained by the one-step sequence- and ligation-independent cloning (SLIC) method described in ref.^35^. All PCR primers employed for plasmid construction are listed in Supplementary Table 3. Genomic DNA was isolated and purified with Pure Link genomic DNA minikit (Invitrogen). PCR reactions for cloning were performed by using Q5® High-Fidelity DNA Polymerase (New England Biolabs, Inc (NEB)) and the products sequenced to verify the absence of any mutation (GATC-biotech).

### Construction of *cntL*, *cntI*, *cntO* and *cntI/L* deletion mutant strains of *P. aeruginosa*

Two DNA fragments corresponding to upstream and downstream regions of *cntL, cntI* or *cntO* genes were amplified from PA14 chromosomal DNA with PCR primers SL1/2 & SL3/4 for *cntL*; SL12/13 & SL14/15 for *cntO* and SL19/20 & SL20/21 for *cntI* (Supplementary Table 3). Upstream and downstream regions were ligated by overlapping PCR and cloned into linearized pKNG101 by the SLIC method. The resulting constructs were transformed into *E. coli* CC118λ*pir* and introduced into *P. aeruginosa* PA14 by conjugation. The strains in which the chromosomal integration event occurred were selected on *Pseudomonas* isolation agar Gm plates. Excision of the plasmid, resulting in the deletion of the chromosomal target gene was performed after selection on Luria-Bertani (LB) plates containing 6% sucrose. Clones that became sucrose resistant and Sm sensitive were confirmed to be deleted for the gene of interest by PCR analysis. The Δ*cntI* Δ*cntL* double mutant strain was constructed by knocking-out *cntL* in the *cntI* mutant strain.

### Construction of *P. aeruginosa* strains with *cntL*_*V5*_ and *cntL* alleles inserted at aat site

DNA fragments corresponding to the *cnt* promoter region (see sequence Fig. 1) and *cntL*_*V5*_ or *cntL* alleles were generated by PCR from PA14 chromosomal DNA with PCR primers SL7/8 & SL9/10 or SL9/50 (Supplementary Table 3). Upstream and downstream regions were ligated by overlapping PCR and cloned by the SLIC method in Mini-CTX1 vector. The resulting plasmid was introduced into *P. aeruginosa* PA14WT and PA14*zur* ^*−*^ strains by conjugation. The recombinant clones containing the mini-CTX1 inserted at the *attB* locus on the *P. aeruginosa* genome were selected on tetracycline-containing PIA. Excision of the unwanted plasmid DNA sequences (genes and associated promoter sequences that might interfere with expression of genes cloned into the MCS) located between the *FRT* sites (present on the mini-CTX1) was achieved by expressing Flp recombinase from a conjugative plasmid, pFLP2 which was introduced into *P. aeruginosa* PA14 by conjugation. The *P. aeruginosa* PA14 clones containing the pFLP2 were selected on carbenicillin-containing PIA. Finally, selection for pFLP2 deficient strains was done after selection on LB plates containing 6% sucrose. Colonies that became sucrose resistant and Cb^S^ have lost the pFLP2 plasmid.

### RNA isolation and RT-PCR reactions

Total RNA was prepared from *P. aeruginosa* strain PA14 mid-log phase cultures grown in MS medium using the SV Total RNA Isolation System (Promega). Contaminating DNA was removed by digestion with Dnase I (RTS Dnase kit - Ozyme). DNA-free total RNA was then used as a template for reverse transcriptase reactions using the Superscript III reverse transcriptase and random hexamers as described by the manufacturer (Invitrogen). Intragenic regions of *cntO, L, M* and *I* (see regions 1 to 4 Fig. 1) were respectively amplified with primer sets SL42/43, SL44/45, SL46/47, SL48/49. Intergenic regions upstream of *cntO*, between *cntO* and *cntL*, between *cntL* and *cntM*, between *cntM* and *cntI* and downstream *cntI* (see regions 5 to 9 Fig. 1) were respectively amplified with primer sets SL38/39, SL32/33, SL34b/35b, SL36/37, SL40/41.

### Protein detection by immunoblotting

PA14∆*cntL::cntL*_*V5*_ was grown at 37 °C in MS medium or Luria-Broth (LB) medium. When optical density at 600 nm (OD_600_) reached 0.4 to 0.6 a volume of culture equivalent to 2 OD_600_ units was centrifuged for 2 min at 2.000 g. The pellet corresponding to whole cells was resuspended in 1X SDS-PAGE loading buffer containing β -mercaptoethanol and heated for 10 min at 95 °C. Proteins samples equivalent 0.1 OD_600_ units were separated by SDS-PAGE. Electrophoresis was performed using 12% SDS-polyacrylamide gel at room temperature and 25 mA/gel. Immunoblotting was performed as previously described^36^, with rabbit primary and peroxidase-conjugated secondary antibodies respectively directed against V5 epitope (Bethyl/interchim) (dilution 1:5,000) and rabbit IgG (Sigma) (dilution 1:25000). The peroxidase reaction was developed by chemiluminescence (Pierce), scanned and analyzed with ImageQuant LAS 4000 camera and TL analysis software (GE Healthcare Life sciences).

### Protein detection by dot blot

A nitrocellulose membrane was incubated 5 min with transfer buffer and dried at room temperature for 3 min. A 5 µL drop of SDS-PAGE protein sample (equivalent to 0.5 OD_600_ units was loaded onto nitrocellulose membranes. After drying, the proteins were transferred on the nitrocellulose for 5 min at 80 V and 0.1 A using a Fast Blotter System (Pierce). Immunoblotting was perform with SNAP i.d.^®^ 2.0 Protein Detection System. The membrane was blocked 5 min in TBS (1X) Tween 20 (0.1%) skim milk (0.5%), washed 4 times with TBS-Tween, incubated 10 min in TBS-Tween-milk with primary rabbit antibody directed against the V5 epitope (dilution 1:5,000), washed 4 times with TBS-Tween, incubated 10 min in TBS-Tween-milk with anti-rabbit peroxidase-conjugated antibody and the peroxidase reaction was developed by chemioluminescence, scanned with ImageQuant LAS 4000 camera and analyzed by the ImageQuant TL analysis software.

### Viability essay on plate

Pre-culture of PA14 strains were performed overnight in MS medium under horizontal shaking at 37 °C. The next day, a culture of fresh MS medium is inoculated by the pre-culture at OD_600_ of 0.1 and incubated for 6 hours under horizontal shaking at 37 °C. Cultures were then adjusted to OD_600_ of 1 and subjected to 10% serial dilutions in fresh MS medium. 10 µl of culture samples were spotted on MS 5% agar plate an incubated overnight at 37 °C.

### Sample preparation for analysis by HILIC/ICP-MS, HILIC/ESI-MS and determination of metal concentrations by ICP-MS

All the *P. aeruginosa* strains derivatives of the parental PA14 strain were grown aerobically with horizontal shaking at 37 °C in freshly made Minimal Succinate (MS) medium. All media used in this study were filtered at 0.22 μm with polycarbonate units before used, and stored at 4 °C away from light in polycarbonate bottles. Pre-culture of 20 mL were inoculated from fresh MS-5% agar plates and grown until late exponential phase in polycarbonate erlenmeyers. Culture of 25 mL were inoculated at OD = 0.1 and grown for 8 h in freshly made MS or CDM media supplemented or not with EDTA or nickel before inoculation. After 8 h, OD_600_ were measured before cells were harvested by centrifugation (2,000 *g*, 30 min, 4 °C). The supernatant was collected, filtered and stored at −80 °C. The pellet was washed twice with 1.3 mL MS media + 1 mM EDTA followed by a wash with 1.3 mL MS media. OD_600_ were measured, and cells ruptured by successive sonication cycles. The lysates were then centrifuged at 16,000 g for 30 min at 4 °C and supernatants were collected and stored at −80 °C. These cell lysate and supernatant fractions were used for analysis of metal complexes using HILIC/ICP-MS and HILIC/ESI-MS as described below. For metal quantitation, growth of WT and mutant strains was done as described above. After 9 h of growth, OD_600_ was measured before cells were harvested by centrifugation (2,000 *g*, 30 min, 4 °C). The pellet was washed 2 times with 1.3 mL MS media + 1 mM EDTA followed by a wash with 1.3 mL MS media. After the OD_600_ was measured, cells were dried overnight at 95 °C. The metal quantification was determined by inductively coupled plasma mass spectrometry as described elsewhere^17^. Relative pseudopaline level was determined using the relative intensity of the m/z corresponding to the pseudopaline-Ni complex in extracellular or intracellular fractions saturated with nickel, in order to force nickel complex formation.

### Analysis of metal complexes using HILIC/ICP-MS and HILIC/ESI-MS

Microbore HILIC separations were performed using an Agilent 1100 capillary HPLC system (Agilent, Tokyo, Japan) coupled either to ICP-MS detection (7500 cs instrument, Agilent) or to an LTQ Orbitrap Velos mass spectrometer (Thermo Fisher Scientific, Bremen, Germany). The column used for HILIC separation was a TSK gel amide 80 (250 mm × 1 mm i.d., 5 μm) from Tosoh Biosciences (Stuttgart, Germany). Gradient elution, at a flow rate of 50 µl min^−1^, was carried out using eluent A, acetonitrile, and eluent B, 5 mM ammonium formate (pH 5.5). Samples were diluted with acetonitrile and water to obtain a 1:2, sample to acetonitrile ratio, and centrifuged. A 7 µl aliquot of the supernatant was injected into the HILIC column each time. To get accurate masses during HILIC/ESI MS analysis, MS and MS/MS spectra were recalibrated offline using precursor/fragment ions with known formula. Putative metal species were fragmented during a subsequent chromatographic run with collision induced dissociation (CID) mode at 35% energy. Signals were recorded at m/z corresponding to pseudopaline complexes with Ni and Zn (C_15_H_21_N_4_O_8_Ni^+^ and C_15_H_21_N_4_O_8_Zn^+^ respectively). Detailed procedures for chromatographic and MS analyses were described elsewhere^17^.

### Statistics

Statistics were determined using the Student’s t-test function of excel using a bilateral model and assuming equal variance.

### Protein cloning, expression and purification

The *cntL* gene of *P. aeruginosa* P14 strain was amplified from genomic DNA and cloned in the vector pET22b^+^ using the NdeI and XhoI restriction sites. This lead to a protein fused with a C-Terminal His_6_ tag. The *cntM* gene of *P. aeruginosa* P14 strain was amplified from genomic DNA and cloned in the vector pET-TEV using the NdeI and XhoI restriction sites. This lead to a protein fused with a N-Terminal His_6_ tag that could eventually be cleaved by TEV protease. Both vectors were transformed into *E. coli* BL21 strains for protein expression. The strain carrying the pET22b^+^ *cntL* plasmid were allowed to grow in LB medium containing 100 µg/ml ampicilline to an OD_600_ of 0.7 before inducing expression with 1 mM Isopropyl-ß-D-thiogalactopyranoside (IPTG) followed by 20 hours incubation at 16 °C. The strain carrying the pET-TEV*cntM* plasmid were allowed to grow in autoinducible medium containing 100 µg/ml ampicilline for overnight growth at 37 °C. In both cases, the cells were pelleted, resuspended in buffer A (50 mM phosphate, 450 mM NaCl, 20 mM imidazole, pH 8.0), and disrupted with a French Press at 7 Mpa. The resulting soluble fraction was loaded on a Nickel charged column (HisTrap column, GE Healthcare) and the protein was eluted by an imidazole step gradient (50 mM wash and 200 mM elution). Gel filtration experiments were done using a Hiload 26/60 superdex200 column (GE Healthcare) using buffer B (50 mM HEPES, 50 mM NaCl, pH7.0)

### Fluorimetry and TLC experiments

Fluorescence resonance energy transfer (FRET) experiments were performed using a Varian Cary Eclipse spectrofluorimeter with an excitation wavelength of 280 nm (tryptophan excitation, emission at 340 nm that is transferred to the NAD(P)H and recording of the emission at 460 nm. Ligand binding was determined from the partial enhancement of this fluorescence emission. The amplitude of the FRET was fitted with a simple binding model using SigmaPlot software. TLC experiments were done as described elsewhere^17^.

## Electronic supplementary material

Supplementary material
