# Supplementary material for: Pseudomonas aeruginosa zinc uptake in chelating environment is primarily mediated by the metallophore pseudopaline
Source: Sci Rep. 2017 Dec 7;7:17132. doi: 10.1038/s41598-017-16765-9 (PMC5719457; doi:10.1038/s41598-017-16765-9)
Supplement: Supplementary file 1 — Supplementary material [file 41598_2017_16765_MOESM1_ESM.pdf]

Pseudomonas aeruginosa zinc uptake in chelating environment is primarily mediated by the metallophore pseudopaline

Sébastien Lhospice[1,§], Nicolas Oswaldo Gomez[1,§], Laurent Ouerdane[2,§], Catherine Brutesco[3], Ghassan Ghssein[3], Christine Hajjar[3], Ahmed Liratni[1], Shuanglong Wang[2], Pierre Richaud[4], Sophie Bleves[1], Geneviève Ball[1], Elise Borezée-Durant[5], Ryszard Lobinski[2], David Pignol[3], Pascal Arnoux[3,*] and Romé Voulhoux[1,*]

[1]CNRS et Aix-Marseille Université, Laboratoire d'Ingénierie des Systèmes Macromoléculaires (UMR7255), Institut de Microbiologie de la Méditerranée, Marseille, France.

[2]Université de Pau et des Pays de l'Adour/CNRS, Laboratoire de Chimie Analytique Bio-inorganique et Environnement, IPREM-UMR5254, Hélioparc, 2, Avenue Angot, 64053 Pau, France.

[3]CEA, CNRS and Aix-Marseille Université, Institut de Biosciences et Biotechnologies d'Aix-Marseille, UMR 7265 LBC, CEA Cadarache, Saint-Paul-lez-Durance F-13108, France.

[4]CEA, CNRS and Aix-Marseille Université, Institut de Biosciences et Biotechnologies d'Aix-Marseille, UMR 7265 LB3M, CEA Cadarache, Saint-Paul-lez Durance F-13108, France.

[5]Micalis Institute, INRA, AgroParisTech, Université Paris-Saclay, 78350 Jouy-en-Josas, France.

[§]Contributed equally to this work

[*]Correspondence E-mail: pascal.arnoux@cea.fr and voulhoux@imm.cnrs.fr

Supplementary material

Supplementary figures and tables

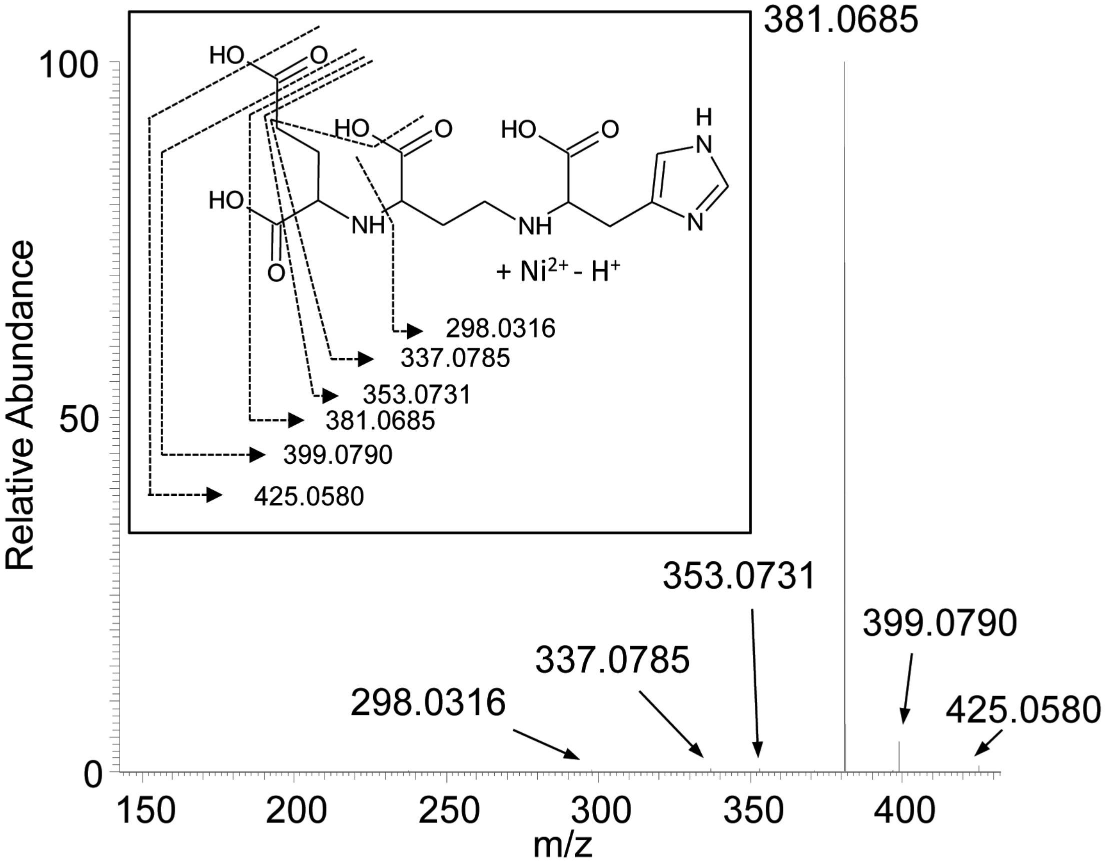

Supplementary Figure 1 | MS/MS fragmentation of Pseudopaline-Ni complex. The mass of the ions are indicated as well as their interpretation with the deduced fragmentation scheme shown in the inset.

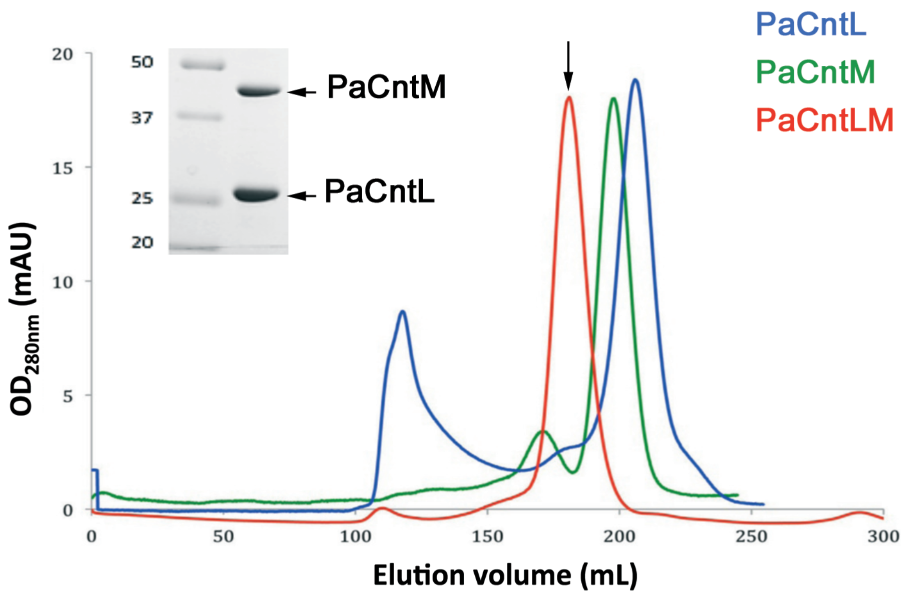

Supplementary Figure 2 | Final purification steps of PaCntL, PaCntM and identification of a complex between PaCntL and PaCntM by gel filtration. The elution profiles of PaCntL (blue trace) PaCntM (green trace) and a mix of PaCntL and PaCntM (red trace) are shown, with a SDS-PAGE gel using the pic fraction of the PaCntLM elution with molecular weight markers indicated on the left (inset).

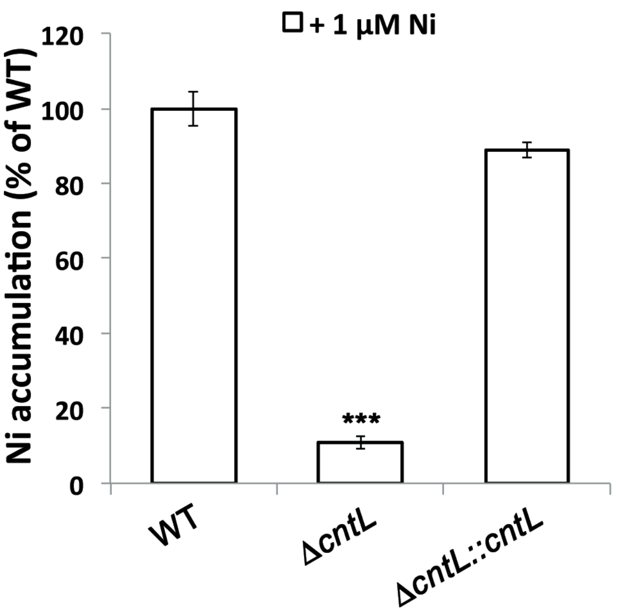

Supplementary Figure 3 | Pseudopaline is involved in nickel uptake in minimal media supplemented with 1 µM nickel. Intracellular nickel levels were measured by ICP-MS in WT, *ΔcntL* and *ΔcntL::cntL* strains grown in MS medium and supplemented with 1 µM of nickel. Error bars, mean ± s.d. **P*<0.05, ***P*<0.01 and ****P*<0.001.

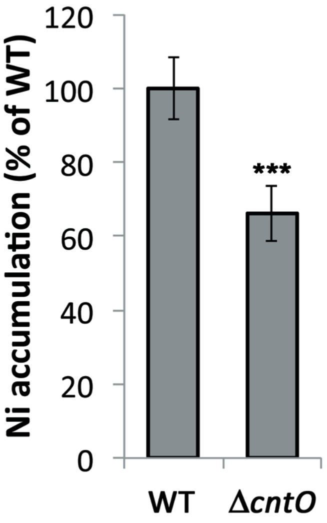

Supplementary Figure 4 | Involvement of PaCntO in the import of Ni. Comparison of the nickel intracellular accumulation in the WT and ΔcntO mutant strains. Error bars, mean ± s.d. **P*<0.05, ***P*<0.01 and ****P*<0.001.

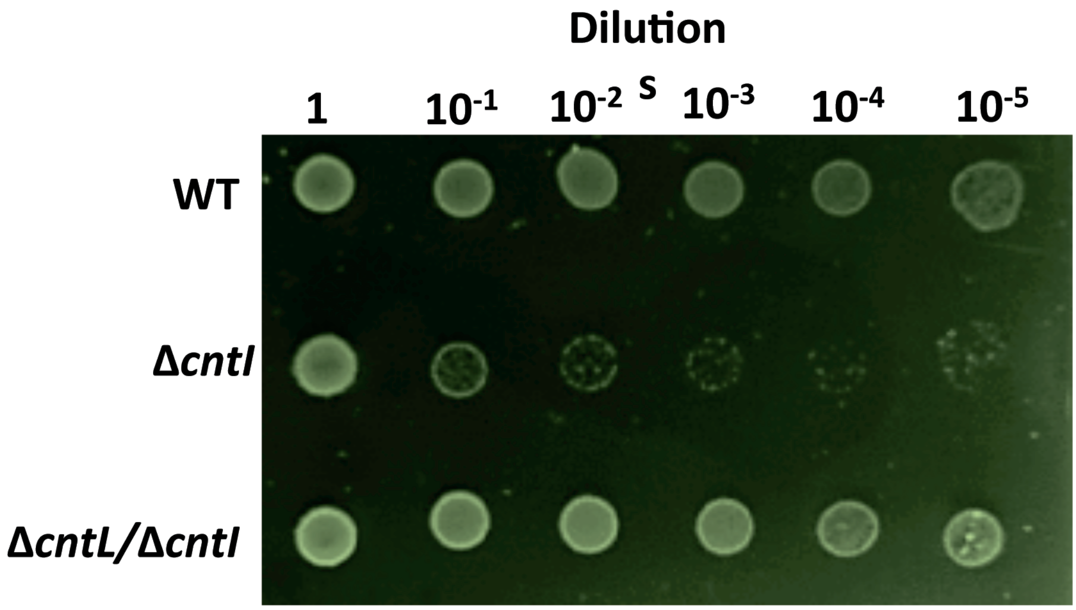

Supplementary Figure 5 | Cell viability assay of WT, Δ*cntI* single mutant and Δ*cntL*/Δ*cntI* double mutant. Cell viability was assessed by serial dilutions of PA14 strain cultures spotted on MS agar plates.

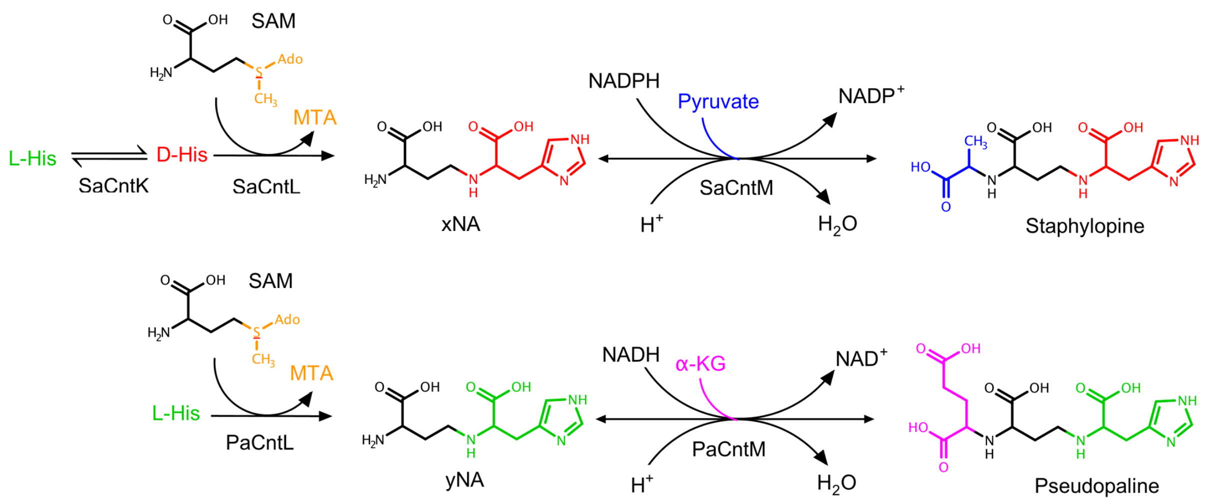

Supplementary Figure 6 | Differences in the staphylopine (top) and pseudopaline (bottom) biosynthetic pathways.

	**Gene name and locus tag**			
**strain**	***cntO***	***cntL***	***cntM***	***cntI***
**PAO1**	PA4837	PA4836	PA4835	PA4834
**PA14**	PA14_63960	PA14_63940	PA14_63920	PA14_63910
**PA7**	PSPA7_5556	PSPA7_5555	PSPA7_5554	PSPA7_5553

Supplementary Table 1 | correspondence with locus tag in PAO1 and PA14 and PA7 strains of *P. aeruginosa.*

	description	Reference
E. coli strains		
CC118λ*pir*	Δ(*ara-leu*) *ara*D Δ*lac*X74 *gal*E *gal*K *pho*A20 *thi*-1 *rps*E *rpo*B *arg*E (Am) *rec*A1 RfR (λpir)	[1]
SM10	*thi*-1, *thr*, *leu*, *ton*A, *lac*Y, *sup*E, *rec*A::RP4-2-Tc::Mu; Km^R	Laboratory collection
BL21	F^- *omp*T *hsd*S_B (r_B^-, m_B^-) *gal dcm ara*B::T7RNAP-*tet*A	Laboratory collection
P. aeruginosa strains		
PA14	Wild type	[2]
PA14Δ*cntL*	*cntL* (*PA14_63940)* deletion mutant	This work
PA14Δ*cntI*	*cntI* (*PA14_63910)* deletion mutant	This work
PA14Δ*cntO*	*cntO* (*PA14_63960)* deletion mutant	This work
PA14Δ*cntL::cntL*$_{V5}$	PA14Δ*cntL* strain with *cntL*$_{V5}$ allele under the control of the *cnt* promoter integrated at the *attB* site (*::cntL*$_{V5}$*)*	This work
PA14Δ*cntL::cntL*	PA14Δ*cntL* strain with *cntL* allele under the control of the *cnt* promoter integrated at the *attB* site (*::cntL)*	This work
PA14Δ*cntL*Δ*cntI*	*cntL cntI* double deletion strain	This work
PA14*zur*$^-$	PA14 strain with transposon insertion in the *zur* gene *PA14_72560* (*zur::Tn* or *zur*$^-$) (mutant ID 42601)	[2]
PA14*::cntL*$_{V5}$ *zur*$^-$	PA14*zur::Tn* (*zur*$^-$) strain with *cntL*$_{V5}$ allele under the control of the *cnt* promoter integrated at the *attB* site	This work
Vectors and Plasmids		
pKNG101	Suicide vector SmR, *oriR6K*, *oriTRK2*, *mobRK2*, *sac*BR+	[1]
pKNG101Δ*cntL*	Suicide plasmid for *cntL* deletion	This work
pKNG101Δ*cntI*	Suicide plasmid for *cntI* deletion	This work
pKNG101Δ*cntO*	Suicide plasmid for *cntO* deletion	This work
Mini-CTX1	vector containing *attP* site for integration at the *attB* site of *P. aerugionosa* chromosome; Tc^R.	[3]
Mini-CTX1-*cntL*$_{V5}$	plasmid harboring *cntL*$_{V5}$ under the control of the *cnt* promoter cloned in *Eco*RI of Mini-CTX1; Tc^R.	This work
Mini-CTX1-*cntL*	plasmid harboring *cntL* under the control of the *cnt* promoter cloned in *Eco*RI of Mini-CTX1; Tc^R.	This work
pFLP2	plasmid harboring the inducible *flp* recombinase; ApR (Cb^R).	[3]
pRK2013	Plasmid for triparental mating. Km^R, ColE1, Tra+ Mob+	[4]
pET22b+	Expression plasmid	Novagen
pET22b$^+$*cntL*	plasmid harboring *cntL*	This work
pET-TEV	Expression plasmid	Addgene
pET-TEV*cntM*	plasmid harboring *cntM*	This work

Supplementary Table 2 | Strains and plasmids used in this study.

Name	Sequence (5’-3’)
SL1	CAGGTCGACGGATCCCCGGGGAAAAAGAAGAACGTGCTCACC
SL2	GGCCTTCTCCATGGCATGGCTTCCTGGCG
SL3	GCCATGCCATGGAGAAGGCCGGTCGATGA
SL4	TATGCATCCGCGGGCCCGGGAGGTAGACCCTGCGCTTGAC
SL7	GCTTGATATCGAATTCGGCTGGGCTGGTCGT
SL8	GTAGAGGGCGGGAAATCGCACCAGAAAAG
SL9	CGATTTCCCGCCCTCTACCGCCGCCAGGA
SL10	CGGGCTGCAGGAATTCTCACGTAGAATCGAGACCGAGGAGAGGGTTAGGGATAG GCTTACCTCGACCGGCCTTCTC
SL12	CAGGTCGACGGATCCCCGGGGAAATGCAGCGGATCGAG
SL13	GAGGGCTCACATGGGAAATCGCACCAGAA
SL14	TTTCCCATGTGAGCCCTCTACCGCCGCCA
SL15	TATGCATCCGCGGGCCCGGGCCTCTTCGTCGATGTCCAG
SL19	CAGGTCGACGGATCCCCGGGTCTACCCGGAGGGACCTATC
SL20	GGAGGCTCACTTCAGCAGGTCGAGCACCA
SL21	CTGCTGAAGTGAGCCTCCGGCGCGACCGG
SL22	TATGCATCCGCGGGCCCGGGGCTGCTCTACAGCATCTCGAC
SL32	ΓΤΧΓΧΤΑΧΤΑΧΓΑΓΧΓΤΤΧΧ
SL33	GATGTCCAGGCAGCACAAA
SL34b	AACTGGAGAAGCACCTTTGC
SL35b	GACCACGTCCAGGTAACTGTC
SL36	GATTCATCGATTGCCAAGGA
SL37	GGAATAGCTGAACGGCTTGA
SL38	GAGCAGCATGAACAGCATCA
SL39	GGATGTCCTCGATACGGGTG
SL40	GCTATATCGGCATCGTCTTCA
SL41	CATGCTCCAGGAGATCAAGC
SL42	TCAGTGTGTCGCTTGTCCTC
SL43	GCTTCTTGGTCACCAGGTTC
SL44	GAGATTCGCCTGCTCACC
SL45	GATGTCCAGGCAGCACAAA
SL46b	ACCAAGGTGATCGACGAGAC
SL47b	CTCCTTGGCAATCGATGAAT
SL48	ATCGGTACCCTGCTGATCTA
SL49	CACCGCCAGGAAGTAGAAGA
SL50	CGGGCTGCAGGAATTCTCATCGACCGGCCTTCTCCA

Supplementary Table 3 | Oligonucleotides used in this study.

Supplementary references

1. Kaniga, K., Delor, I. & Cornelis, G. R. A wide-host-range suicide vector for improving reverse genetics in gram-negative bacteria: inactivation of the blaA gene of Yersinia enterocolitica. *Gene* **109,** 137–141 (1991).

2. Liberati, N. T. *et al.* An ordered, nonredundant library of Pseudomonas aeruginosa strain PA14 transposon insertion mutants. *Proc. Natl. Acad. Sci. U. S. A.* **103,** 2833–2838 (2006).

3. Hoang, T. T., Kutchma, A. J., Becher, A. & Schweizer, H. P. Integration-proficient plasmids for Pseudomonas aeruginosa: site-specific integration and use for engineering of reporter and expression strains. *Plasmid* **43,** 59–72 (2000).

4. Figurski, D. H. & Helinski, D. R. Replication of an origin-containing derivative of plasmid RK2 dependent on a plasmid function provided in trans. *Proc. Natl. Acad. Sci. U. S. A.* **76,** 1648–1652 (1979).
